# COVID-19 memes going viral: On the multiple multimodal voices behind face masks

**DOI:** 10.1177/0957926520970385

**Published:** 2021-03

**Authors:** Marta Dynel

**Affiliations:** University of Łódź, Poland; Vilnius Gediminas Technical University, Lithuania

**Keywords:** Echo, epistemological ambiguity, intertextuality, meme, multimodal humour online, parody, participant role, playful trolling, virality, voice

## Abstract

Advancing the concept of multimodal voicing as a tool for describing user-generated online humour, this paper reports a study on humorous COVID-19 mask memes. The corpus is drawn from four popular social media platforms and examined through a multimodal discourse analytic lens. The dominant memetic trends are elucidated and shown to rely programmatically on nested (multimodal) voices, whether compatible or divergent, as is the case with the dissociative echoing of individuals wearing peculiar masks or the dissociative parodic echoing of their collective voice. The theoretical thrust of this analysis is that, as some memes are (re)posted across social media (sometimes going viral), the previous voice(s) – of the meme subject/author/poster – can be re-purposed (e.g. ridiculed) or unwittingly distorted. Overall, this investigation offers new theoretical and methodological implications for the study of memes: it indicates the usefulness of the notions of multimodal voicing, intertextuality and echoing as research apparatus; and it brings to light the epistemological ambiguity in lay and academic understandings of memes, the voices behind which cannot always be categorically known.

## Introduction

The worldwide COVID-19 pandemic in 2020 has monopolised news reports and public discussions in traditional media and on social media. Both experts and non-experts publicly express their views about the new coronavirus and safety measures applied to curb its spread. Media reports cause confusion and fan public panic by sharing conflicting beliefs and assumptions, based on which citizens develop their opinions. Especially during the first weeks of the pandemic, stores were running low on supplies, ranging from food to toilet paper, soap and sanitisers, as well as face masks. The best part of the year has seen cities and countries being put on lockdown and civilians being quarantined or given stay-at-home orders/recommendations for the sake of their own safety.

All this stimulates citizens’ social media activity, which shows in their prolific production of *digital humour*, commonly called ‘memes’, about COVID-19. This productivity is reflected in the plethora of reports on online platforms, which recognise the phenomenon and present innumerable lists of ‘best of’ COVID-19 memes. Many non-academic reports refer to the soothing and uplifting role that such humour performs.^1^ Indeed, *relief* is a well-documented psychological function of humour, which can serve as a coping strategy, especially in tragic circumstances (see e.g. Martin, 2007; Martin and Ford, 2018 and references therein). Humour is used as a collective defence mechanism for the sake of ‘mental hygiene’ (Dundes, 1987), as well as *solidarity* building, which is pronounced in online humour about tragedies and crises (e.g. Demjén, 2016; Dynel and Poppi, 2018, 2020). Humour is capable of reframing the source of negative experiences and/or emotions (such as suffering, anxiety and fear) as a source of positive emotions, bringing users psychological relief, at least temporarily (cf. Kuiper et al., 1993; Martin, 2007).

Since COVID-19 humour co-exists with daily reports on the death toll and infection numbers, it may be thought of as *dark humour* (Bischetti et al., 2020), that is humour about – or, at least, inspired by and produced in the context of – grave events and topics, notably, death and illnesses (see Dynel and Poppi, 2018 and references therein). Even though the appreciation, that is funniness versus aversiveness, of COVID-19 humour must rely on various factors, one of them being the distance from the epicentre (Bischetti et al., 2020) and the very nature of a given specimen (e.g. whether or not it addresses the topic of death or disease per se), the prevalence of this humour on social media indicates its social significance.

**Figure 1.**
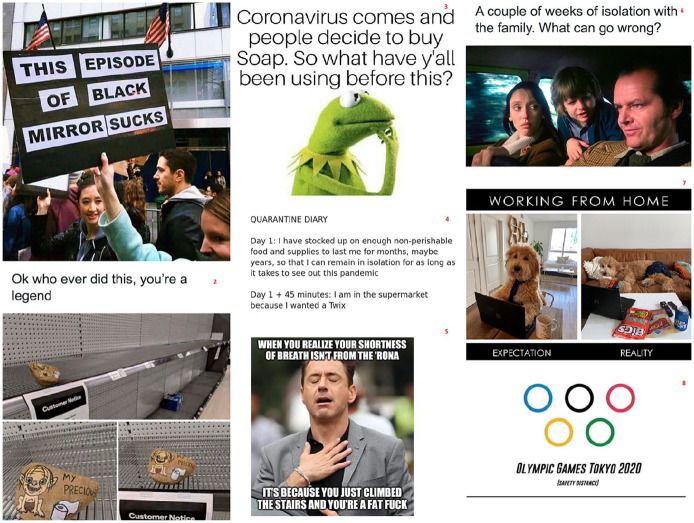
Radom COVID-19 memes.

Through posting humorous memes, individual users contribute to *polyvocal*, that is public, discussions on socio-political topics, airing their views on the current events (see Dynel and Poppi, 2020; Milner, 2016; Ross and Rivers, 2018). Therefore, memes can provide insight into current social and political issues, being vessels for public sharing of serious information and opinions (e.g. Al Zidjaly, 2017; Huntington, 2015). The diversity of COVID-19 topics is tremendous, as the sampled memes (based on a ‘#COVID-19 meme’ search on Twitter, 9gag and Reddit) in Figure 1 illustrate.^2^

The COVID-19 memes in Figure 1 show various timely topics such as: the preposterous, unbelievable situation (Example 1); the need to keep social distance, which has a bearing on public events (Example 8); people’s (irrational) drive to stockpile toilet paper, soap and other supplies at the time of crisis, which brings out their vices and foibles (Examples 2–4); and the consequences of the protracted stay at home, such as obesity (Example 5), family issues (Example 6), and a relaxed attitude to work (Example 7). These examples anticipate the central topic of this paper, indicating the multiplicity of perspectives, conceptualised as Bakhtinian *voices*, which are manifest in how the memes are structured.

In the textual meme taken from a humorous quarantine diary (Example 4), the author reports on his/her irrationality; he/she admits to having accumulated a surplus of supplies only to follow a sudden whim, which makes the stay-at-home resolution futile. Example 7 demonstrates a mismatch between the real versus expected work environment at home. The dog embodies what the meme creator sees as a typical home office worker, who – instead of sitting at a desk, neatly dressed – is slouched on the sofa in underwear with snacks strewn around the laptop. In turn, Example 8 displays a modified version of the Olympic Flag with the five rings being separated, rather than interlaced, in order to metaphorically represent the contemporary adage about the need to keep a safe distance. While speaking their own voice, each of the users echoes the anonymous collective voice of the contemporary society with similar experience and views. The same holds for the remaining examples, which explicitly use recognisable voices, including intertextual references to popular culture.

Examples 3 and 6 allude to media artefacts. In the former, Kermit the Frog from *The Muppet Show* and *Sesame Street*, the meme author’s mouthpiece, is pondering on what people had used for hygiene before the coronavirus outbreak, which is responsible for the high demand for soap. Example 6 showcases a still from *The Shining*, a famous psychological thriller in which the possessed husband/father strives to murder his family in an isolated hotel. This meme also features a caption that may be interpreted as dramatic irony, with the characters being blissfully oblivious to what will happen, or as the meme creator’s use of the figure of irony (cf. it is patently obvious that staying home with one’s family for weeks is bound to cause interpersonal problems). Through this intertextual reference to the film, the meme creator hyperbolically comments on the fact that tempers get frayed when family members are made to spend too much time together. On the other hand, Example 5 deploys the popular meme template, known as ‘relief’ or ‘this moment when’, which features Robert Downey Jr. expressing his gratitude for fans in-between takes on the set of *The Judge* (Dedham, MA on 12th July 2013). The actor’s original expression of gratitude is re-conceptualised by the meme creator as a representation of contextualised relief, as evidenced by the caption; it is a relief to know that a potential symptom of the formidable coronavirus infection is actually the result of obesity and lack of exercise.

While these last three memes centre on mass-media intertextuality, some of the examples rely on more complex structures of nested voices. The protest sign in Example 1 that reads ‘This episode of Black Mirror sucks’ and the picture of the rally date back to President Trump’s election victory in 2016. Through the reference to the acclaimed *Netflix* series, the author of the sign observed the similarity between the surreal (and yet strangely familiar) dystopian world depicted in *Black Mirror* and the US election results. The aptness of this metaphorical representation must have been endorsed by the photographer who took the picture, and individuals who (re)posted it online. As the image is reposted with reference to the pandemic, a similar metaphorical parallel is made between the dystopia in *Black Mirror* and the COVID-19 reality. Example 2, in turn, shows an empty shop shelf, with all toilet paper bought out. Someone has jocularly placed a stone with a drawing of a crouching creature and a roll of toilet paper, in tandem with a sign saying ‘My precious’. This is reminiscent of the famous scene from *The Lord of the Rings* featuring Gollum in possession of the precious ring. The meme author thus reports and praises the innocuous prank witnessed in a shop.

Situated in the context of the Bakhtinian notion of voicing and related notions, this study argues that memes about COVID-19 face masks programmatically involve various nested vantage points, whether endorsing or ridiculing previous voices (sometimes through parody), and whether conforming with or recontextualising and re-purposing the original posts as they are reposted, not infrequently to the extent of going viral. The different constellations of multimodal voices help understand the various trends in memes about masks for COVID-19 that emerge from the manually built corpus, indicating how online users create humour about face masks amid the pandemic. Based on this, a new theoretical contribution to humour research is made with regard to the multiplicity of participant roles and multimodal voices involved in memetic humour production. Additionally, the study points to potential epistemological problems in the understanding of memes.

This paper is divided into five sections. Following this introduction, the section entitled ‘Voice, intertextuality, echo and (online) humour’ presents the notion of voicing and proposes broadening its scope to cover *multimodal* discourse, besides briefly reporting on two related notions, namely *intertextuality* and *echoing*. Their previous applications in (online) humour research are also surveyed, and the relevant categories of humour are briefly described. The next section ‘Methodology’ depicts the socio-political context and provides specifics about the data collection method and analysis. This is followed by an examination of the dominant memetic trends in the corpus, with the focus of interest being the nature of the voices involved (‘Analysis: Memetic trends among COVID-19 mask memes’). The article concludes with ‘Discussion and final comments’.

## Voice, intertextuality, echo and (online) humour

Bakhtin (1981 [1975]) proposed the notions of a *voice* and *voicing*, among other things, in the context of the ‘dialogism of the word’ (p. 279) to suggest that language users – even when not quoting verbatim – borrow and merge the words of others. Cooren and Sandler (2014) summarise this conceptualisation as follows, ‘When we speak we *orchestrate* these different voices in our utterances to make them express our own intentions’ (p. 228). Whatever people say is a tacit or overt replication of what they have heard before. Overall, voicing captures the multiplicity of voices and intentions, often referred to as *polyphony* (e.g. Baxter, 2014; Cooren and Sandler, 2014), underlying one’s (spoken or written) words, or – as is postulated here – any form of expression, not only verbal but also non-verbal and even *multimodal*, namely based on multiple integration of meaning-bearing resources across modes (Jewitt et al., 2016; Kress, 2010; van Leeuwen, 2004).

It is perhaps the conscious and/or recognisable replication of previous messages that is most naturally amenable to consideration as voicing. This is also how *intertextuality* is often understood. Nevertheless, as originally put forward, similar to voicing, intertextuality is a much broader concept. Kristeva (1980 [1967]) states that *any* text is ‘a mosaic of quotations’ and ‘the absorption and transformation of another’ (p. 66). However, more narrowly, intertextuality can be thought of as allusions to previous texts – typically cultural artefacts (see Allen, 2000; D’Angelo, 2009) – that receivers must recognise and/or attribute to the authors/sources for the allusions to be effective (cf. the films and characters in the examples in Figure 1). This is how intertextuality is employed in this paper, in line with previous humour studies (e.g. Norrick, 1989; Tsakona, 2018 ; for a recent overview, see Tsakona and Chovanec, 2020), also those about memes (e.g. Dynel and Poppi, 2020; Milner, 2016; Wiggins, 2019).

*Memes*, shorthand for humorous Internet memes, are defined as humorous, multimodal, user-generated ‘digital items sharing common characteristics of content, form, and/or stance’, which are ‘created with awareness of each other’ and ‘circulated, imitated, and/or transformed via the Internet by many users’ (cf. Huntington, 2015: 78; Shifman, 2013: 41). In practice, not all of these conditions need to be met for an item to be called a meme, both in popular parlance and in academic discourse. Essentially, the label ‘meme’ tends to be used for any specimen of online humour, especially if multimodal. Importantly, memes are typically regarded as involving constant modification/transformation, which is what distinguishes them from *viral*s, which spread across digital media in an unchanged form (e.g. Dynel, 2016a; Shifman, 2013; Wiggins, 2019). Presumably, the prototypical form of a humorous Internet meme is a stock image drawn from popular culture, which may be thought of as an intertextual component (see Example 5, Figure 1), combined with a novel caption or a phrase superimposed on the image (see e.g. Dynel, 2016a; Smith, 2019; Wiggins and Bowers, 2015). However, like anything about COVID-19, mask memes can go viral, being repeatedly reposted across social media (whether individually or in ‘best of’ lists), while pre-existing multi-purpose meme templates (e.g. Example 5 in Figure 1) are rarely deployed.

Similar to intertextuality, the Bakhtinian notion of voicing – in the narrower sense, that is as recognisable voices – has been used in humour studies, notably regarding social media, namely creative Tumblr posts (Vásquez, 2019; Vásquez and Creel, 2017), Amazon review parodies, and novelty Twitter accounts (Vásquez, 2019), all of which are shown to creatively blend different voices (whether real, fictional or imagined) in one text. Importantly, Bakhtin’s (1981 [1975]) notion of *double-voicing*,^3^ which means that ‘in one discourse, two semantic intentions appear, two voices’ (Bakhtin, 1984 [1963]: 189), is used to capture the cases where ‘one of these voices is used to mock, or parody, the other’ (Vásquez and Creel 2017: 68). Indeed, as Vásquez (2019) puts it, parody necessarily rests on ‘double voicing, wherein the author adopts a second voice that exaggerates, critiques, ridicules, interrogates, or otherwise polemicizes the first voice. In this way, parodists create “the image of another’s language and outlook on the world, simultaneously represented and representing” (Bakhtin, 1981, p. 45)’ (p. 128). The parodied representation is humorously flaunted (Rossen-Knill and Henry, 1997). Parody is a special type of *imitation* which involves negative evaluation of the imitated target (Lempert, 2014). Thus, the voice alluded to becomes the target of ridicule and/or criticism (Baxter, 2014), and the butt of the humour. Overall, parody necessarily involves users’ taking on the voices/perspectives of others (Vásquez, 2019), who may be specific individuals or an unspecified collective but whose imitated voice must be recognised for the parody to succeed communicatively.

Parody may then be conceptualised as a type of dissociative echoing. The notion of *dissociative echoing* of a representation (a thought or an utterance) is known in pragmatic research as the hallmark of irony from a relevance-theoretic perspective (for a good overview, see Piskorska, 2016). On this view, the figure of irony involves echoing a prior utterance or an unexpressed thought/belief/expectation in order to indicate one’s *dissociative attitude* towards it. Generally, through an echo, which does not need to be ironic, an individual expresses an attitude (whether negative or positive) to a representation alluded to and attributed to someone else, whether or not identifiable (Diez-Arroyo, 2018).

While necessarily involving a dissociative attitude, the *parodic echo of a voice*, whether or not its source is identifiable, should be distinguished from ironic echoing proposed by relevance theoreticians. First, a parodic echo does not involve meaning reversal to implicitly communicate the opposite propositional meaning – as irony does in one way or another – but necessarily relies on hyperbole and/or absurdity, which is optional for irony (on irony, see Dynel, 2018 and references therein). Second, parodic echoing (e.g. in memes or stand-up comedy) may be realised non-verbally and carry no propositional meaning. By contrast, while the figure of irony can be communicated through non-verbal means of expression, which can be paraphrased verbally, it needs to carry verbal propositional meanings. Third, irony – even when having humorous potential – needs to communicate non-humorous negative evaluation (Dynel, 2018) of the echoed thought. Conversely, while the parodic echoing of a voice may serve serious critique (see Dynel, 2020; Hutcheon, 1985; Rose, 1993), in principle, it does not have to communicate any serious, critical meanings outside the *playful, humorous frame* à la Bateson (1972 [1955]; see Dynel, 2017, 2018) and may be done ‘just for fun’ (see also Dentith, 2000; Rose, 1993). Overall, for instance, parodying someone’s gait or facial expression need not entail any serious criticism (and does not carry any propositional meaning); instead, it may merely be a performance based on exaggeration of some salient features solely for the sake of humour and entertainment. This observation is relevant to some COVID-19 mask memes.

## Methodology

Face masks have been one of the hotly debated topics since the news about the new coronavirus started spreading. There have been many theories and claims about whether or not one has to wear a face mask in public and, if so, of what kind in order to protect oneself and/or others from COVID-19. This has caused many diverse reactions, from refusing to wear any face coverings to voluntarily wearing handmade masks (sometimes covering the whole face, including the eyes), presumably with an intent to fully protect oneself from the virus. Also, due to the dearth of medical masks and the high prices of professional anti-viral masks in the first half of 2020, people resorted to various substitutes. The current international consensus – supported by medical evidence – is that while a face mask may not prevent a healthy person from getting the virus, it can prevent a COVID-19-positive person from spreading the virus in social settings if it covers both the mouth and the nose.^4^ Professional anti-viral masks or, at least, cloth face coverings are officially recommended in most countries in community settings. In many countries, masks have been not just recommended but obligatory and must be worn everywhere, also in open spaces. This is the socio-political backdrop against which memes about face masks have been spreading online from January 2020.

The data for the present study were collected systematically in the span of four months, between January and April 2020 (the period when the information about the new coronavirus in China developed into the news about the worldwide pandemic) from 9gag, Reddit, Imgur and Twitter. The searches done at intervals were based on a combination of relevant tags serving as users’ metapragmatic evaluations: ‘#COVID19’ (or ‘#coronavirus’), ‘meme’ and ‘face mask’. This move naturally decreased the number of items to be included in the corpus (making it amenable to manual analysis) since only some memes about masks on the websites bore the three tags. Additionally, in order to be included in the corpus (making it smaller still), the images had to be tagged ‘funny’ or ‘humo(u)r’ and/or be evaluated by at least three users as amusing, as evidenced by their verbal and/or non-verbal reactions, such as emoticons, in the ensuing comments. This was done to avoid the researcher’s bias, a frequent problem in humour studies (for discussion, see Dynel, 2018). In other words, the use of metapragmatic evaluations of humour eliminated the problem of the researcher’s personal (idiosyncratic) perception of humour, which might have warped the results. Also, for simplicity, even though the data sources included items in several meme formats, GIFs and short videos were omitted in the data selection procedure. As the data were being collected, numerous duplicates (i.e. the very same items reposted with no modifications, such as a new title or trimming of the image, the presence of which testifies to the virality of the memes) were eliminated. This complex procedure yielded a manageable dataset of humorous multimodal items (*n* = 174) about COVID-19 masks captured as PNG files. Thus, the variously configured multimodal components fall into visual and textual categories.

These data, that is multimodal memes (cf. Yus, 2019), were duly analysed qualitatively based on a grounded-theory approach and following the premises of Multimodal Discourse Analysis; this involved paying attention to multiple, very subtly interwoven components merging various modalities (e.g. Jewitt et al., 2016; Kress, 2010; van Leeuwen, 2004; Wang, 2014). Importantly, through their choices across modalities, meme authors make some components of meaning more salient while suppressing others (Smith, 2019). Memes are also conducive to complex meaning-making in the light of the relevant socio-political and cultural context (see Dynel and Poppi, 2020; Hakoköngäs et al., 2020; Jiang and Vásquez, 2019; Milner, 2016; Ross and Rivers, 2018; Smith, 2019). Overall, situated in the pertinent socio-cultural context, the memes in the dataset were examined for their multimodal content, as well as had their online history traced, all in line with the critical discourse analytic tradition, where micro- and macro-levels of social structure are pertinent (e.g. van Dijk, 1993).

## Analysis: Memetic trends among COVID-19 mask memes

The empirical part of this study seeks to distil the prevailing trends among the humorous memes about masks. The memes are conceptualised as involving combinations of different (non)humorous voices, whether harmonious or conflicting. An initial analysis of the items in the corpus leads to an observation that, rather than being based on stock images or meme templates (e.g. Dynel, 2016a; Smith 2019; Wiggins and Bowers, 2015), an overwhelming majority of the memes in the corpus centre on photographs of people wearing COVID-19 masks, which can duly go viral or be used as memetic bases.^5^ Therefore, it is useful to draw a distinction between several participant roles involved in the diachronic meme production process: the person in the post, that is the *(meme) subject*; the person who has taken the picture and first published a given meme,^6^ that is the *(meme) author*; and the person who has transformed a meme into a new one, that is the *(meme) poster*, or has merely reposted it with no modification, that is the *(meme) reposter*. The subject and author may coincide in one person (the relatively rare situation when the author can be determined) or be collaborators, thus speaking in one voice. However, on other occasions, their voices may be incompatible, as is often the case with *(re)poster* voices, capable of changing the first meme author’s or the previous poster’s intent (see also Dynel, 2020).

**Figure 2.**
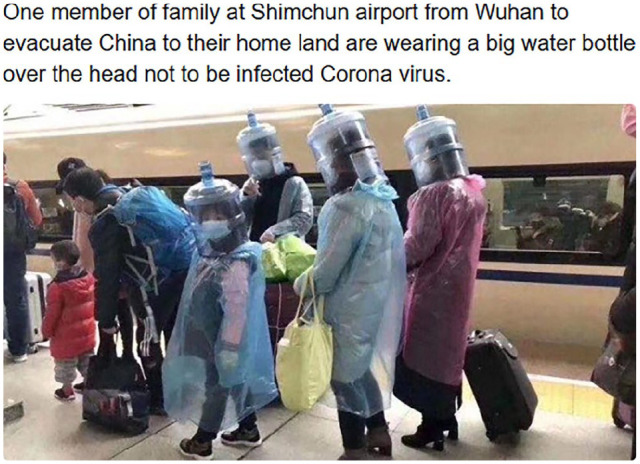
Early tweet about Chinese people’s protective measures.

Memes about COVID-19 masks seem to have originated in recontextualised (Bauman and Briggs, 1990) serious reports on Chinese passengers voluntarily clad in self-made protection, such as the one in Figure 2. This tweet documents a family wearing empty water bottles on their heads and plastic coats, determined not to contract the airborne virus while queueing for a train. Neither the voice of the subjects nor the voice of the tweeter should be taken to display any humorous intent. Nonetheless, this and numerous similar images (without the tweeted text like the one in Figure 2) of people with water bottles and/or wrapped in all kinds of plastic attire were (re)posted as specimens of humour, namely *unintentional humour* from the subjects’ vantage point. This was when the threat of COVID-19 was not yet imminent outside China, and little empathy could be felt for the affected citizens. Thus, their desperate attempts at protecting themselves seemed preposterous and possibly funny when viewed from the safe outsider perspective (cf. Bischetti et al., 2020).

**Figure 3.**
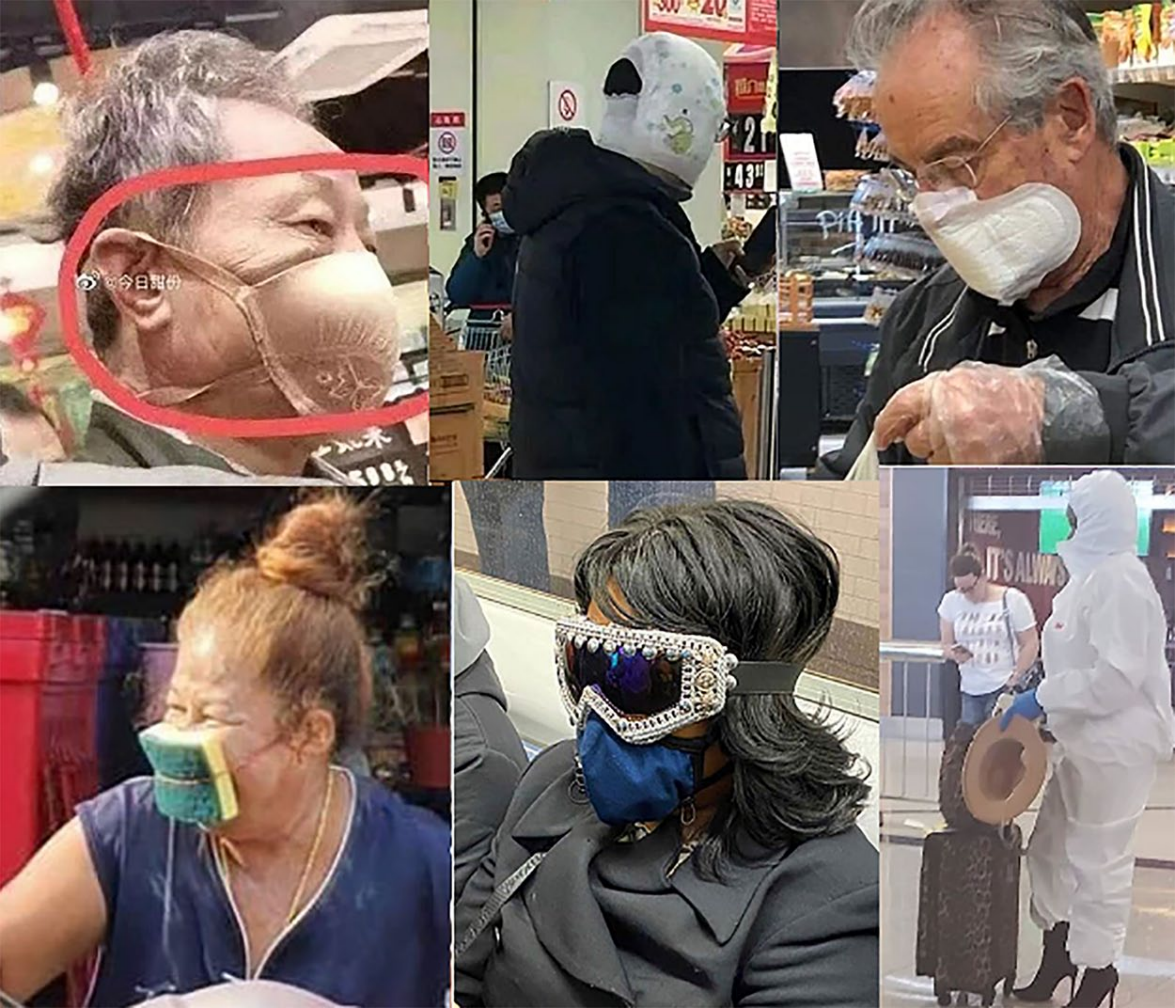
Homemade masks worn sincerely.

It is the dearth of face masks (especially at the beginning of the coronavirus outbreak) and the mongering of fear by the media that appear to have made people seek alternative protection measures, which may be considered amusing given the standard applications of the items used for protection and/or the incompatibility of the attire (see Figure 3). Images like these can induce humorous reactions based on the *incongruity* understood as a cognitive surprise/clash with the receiver’s prior beliefs/expectations (see e.g. Forabosco, 2008; Martin, 2007). Enterprising though they may be, the subjects are pictured as the butts of whom to make fun given the protective solutions of their choice.

The close-up pictures in Figure 3, all taken in public places (in shops/supermarkets, on a train, and at an airport) – presumably, in a stealthy manner – show the meme subjects wearing rather peculiar items on their faces. While the subjects in the images are highly unlikely to have had any humorous intention, the meme authors and (re)posters must have recognised the humour in the peculiar (albeit sometimes canny) safety measures. These include items related to taboo spheres or activities (a padded bra, nappy-pants, and a winged sanitary towel) or simply bizarre solutions, such as a kitchen sponge or fancy ski goggles for eye protection. Yet another item presents a woman dressed in a white plastic suit and blue gloves, which come together with her high heels, hat and leopard-patterned bag-and-suitcase set, all for a fashionable look. In all these cases, the meme authors and (re)posters dissociatively echo, and thereby ridicule, the voices of the meme subjects (butts) multimodally quoted in the pictures.

**Figure 4.**
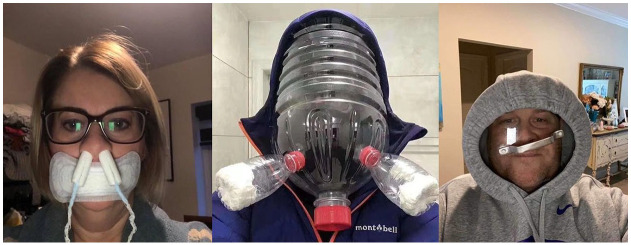
Mask parodies.

A distinct meme trend based on dissociative echoing is illustrated in Figure 4.

**Figure 5.**
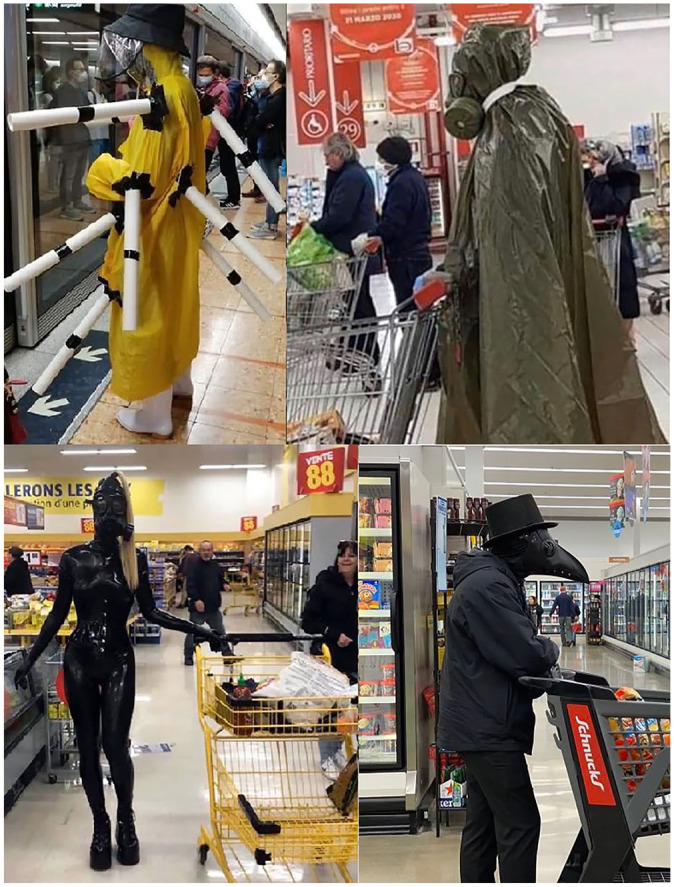
Pranks.

Whether taken as selfies (e.g. Veum and Undrum, 2018) or captured by their collaborators, the three subjects in Figure 4 must have posed for the pictures, sporting their inventions in front of the camera. The woman in the first picture has not only a panty liner glued to her mouth but also two tampons stuck in her nostrils. The subject in the viral picture in the middle is wearing a complicated structure of three plastic bottles and some filtering material. Finally, the subject on the right is one example of the meme series based on people securing saucepan lids in front of their faces by tightening their hoodies around them. Given the context (private, closed spaces, where masks are otiose), the close shots and the evident absurdity, the three ‘masks’ are not to be understood as homemade safety measures that the subjects sincerely endorse. These are rather humour-oriented parodies of masks, such as those worn by the subjects in Figures 2 and 3, whose collective voice is dissociatively echoed through parodic imitation done for fun by individuals stranded at home. The playfully parodic nature of these absurd solutions can only be appreciated when the characteristics of those sincerely worn homemade masks are recognised, as is the exaggeration differential between the originals and the humorous copies (cf. Lempert, 2014). Similar parodic intent may be sought in another meme trend found in the corpus (see Figure 5).

The subjects in Figure 5 are dressed in most peculiar, attention-grabbing attire: a plastic face shield and a coat with poles to guarantee social distance (top left), a plastic cape and an army gas mask (top right), another army mask and a Dominatrix-style latex outfit, as well as a plague doctor’s beak-like mask.^7^ At first blush, unlike the subjects in Figure 4, those in Figure 5 appear not to coincide with the meme authors, let alone posters. Similar to the memes in Figure 3, the pictures show public places (a railway station and supermarkets), purportedly representing a stealthy bystander position as if the peculiar attire is sincerely worn for protection. However, the photographs of the subjects could actually be taken by their accomplices, whoever the meme authors may be. In either case, the subjects may be considered the authors of innocuous *pranks* played on passers-by and shoppers in order to surprise and entertain them (notice the amused woman in the bottom-left meme). The outlandish clothes seem to be worn not for the sake of genuine protection but for the sheer fun of it, or for the sake of dissociative imitation of anti-coronavirus measures or ridicule of the need to take any protective measures. Alternatively, the strangely clad individuals may have been fabricated through editing software, like the beaked mask,^8^ with the (re)posted pranks being intended only for online receivers, who will be amused but – unless in the know – can be deceived that the pictures are entirely genuine. Thus, such pranks may coincide with playful *trolling* done for the sake of amusement (see Dynel, 2016b). However, there is no way of categorically knowing which of the alternative scenarios is/are really true in each case. Nor is it possible to tell whether the meme subjects, authors, as well as (re)posters, have the same voice or whether their voices diverge.

**Figure 6.**
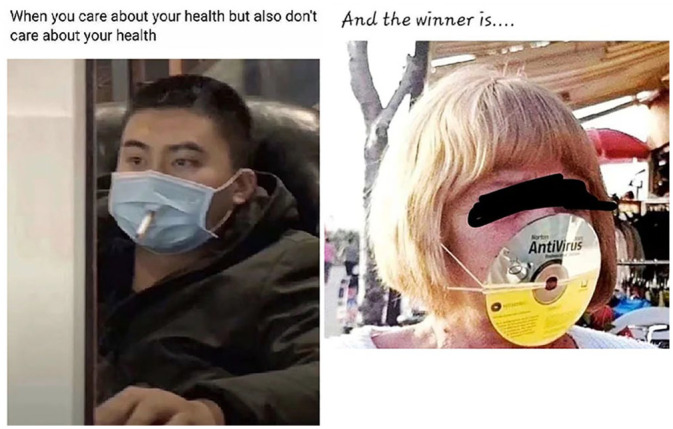
Spoofs (re)posted as sincerely worn masks.

Interestingly, some (re)posters consider memes to multimodally represent the meme subjects’ serious voices, sometimes criticising the attire for its inefficiency. Such criticism has been levelled at the subjects in the two memes in Figure 6,^9^ both (re)posted and interpreted as specimens of sincerely performed human activities witnessed in public spaces. These two memes signpost an important epistemological problem, which analyses of memes should account for.

**Figure 7.**
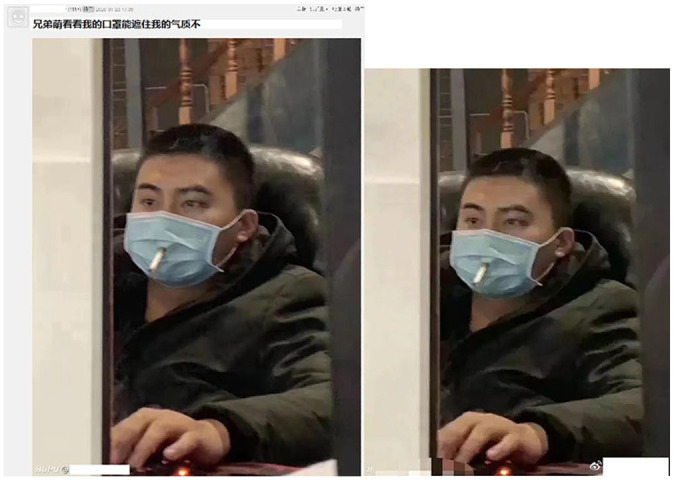
Previous versions of the smoker meme.

The image of the Chinese man smoking through a hole in his mask – which makes the mask useless – may be interpreted as a picture taken by stealth to show the subject as the unknowing butt of whom to make fun (cf. Figure 2). The dissociative voice criticises not only the man’s inefficient protection against the coronavirus but also his jeopardising his health by smoking, as the header indicates (the topic of many metapragmatic comments about this meme). This subject-as-the-butt interpretation of the viral image (often reposted without the caption seen in Figure 6), however, invites serious doubts. Even though the man is wearing a parka, the picture seems to have been taken indoors, as the man is sitting in a leather armchair, which makes it very unlikely to be any indoor public space, where smoking is disallowed in China.^10^ A multi-stage online search through Google images has yielded hundreds of other specimens of this picture. While it is impossible to trace the original source, many of the memes date back to 23rd January 2020 and can be found on Chinese websites (see Figure 7). The previous memes indicate that the original image must have been trimmed (partly to hide the embedded user names). These broader-view images testify that the man is in a house (cf. the bannister behind him) and that, with his hand on a keyboard, he must be gazing at a computer screen.

The meme on the left found on Hupu, a sports commentary and news platform, seems to be centred on a selfie with a header indicative of the subject’s voice, ‘Hey, guys/brothers, do you think the mask can hide my charming looks?’ The meme poster’s nickname (deleted here) is the same as the one embedded in the bottom-left corner of the picture, suggesting the authorship of the image. On the other hand, the meme on the right comes from Sina Weibo, the Chinese Twitter counterpart. This is an evident repost, with the obliterated user name and the original platform name (cf. the still visible ‘H’ in the bottom left-hand corner) and a new user name added on the right (withheld here). Interestingly, the meme on the right cannot be a modification of the one on the left insofar as the latter was posted a few minutes later. Overall, whatever its provenance and original voice may be, this smoker image has snowballed across English-speaking social media and is already known as the ‘outbreak smoke brake’ meme template.^11^

**Figure 8.**
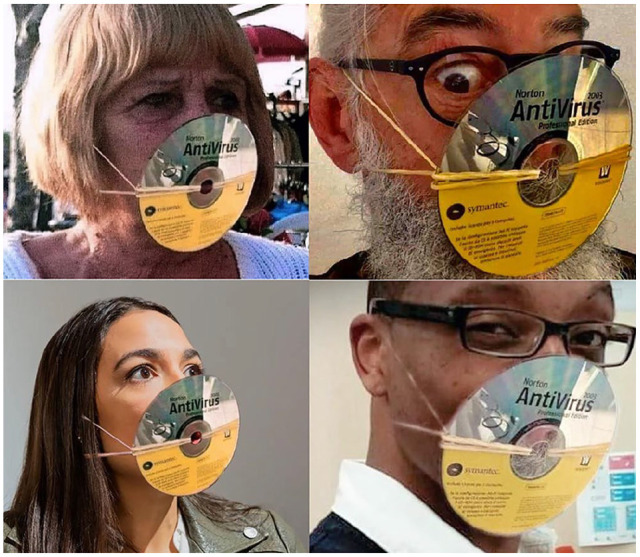
Examples from the AntiVirus meme series.

The story behind the second meme in Figure 6 showing the woman with the AntiVirus software CD on her face is clear-cut. The poster of the meme bearing the header ‘And the winner is. . .’ (indicative of, allegedly, the most inadequate protection against the coronavirus) purports to submit it as a real specimen spotted by the poster. This impression is strengthened by obliterating the woman’s eyes for the sake of preserving her anonymity. However, this meme bears visible markers of being photoshopped (notice the quality of the lines representing the rubber along the woman’s cheek). This is a version of a previous meme, which the poster must have encountered (see the first meme in Figure 8) only to modify it. It is difficult to tell beyond any doubt whether the meme poster’s reuse of the image is a purposeful attempt at trolling (see Dynel, 2016b) in order to deceive the receivers into believing that the subject’s voice is genuine; or whether the poster, ignorant of the humorous meme series and the collective voice behind it (see Figure 8), naïvely believed the encountered viral picture to demonstrate genuine mask-wearing.

The memes in the series in Figure 8, presumably commenced by the second image (as evidenced by the date of posting and the quality of the photograph), capitalises on the pun couched in the polysemy of ‘virus’, which may refer to the cause of an infectious illness or a set of instructions making a computer malfunction. This memetic spoof series features faces with 2003 AntiVirus CDs (an obsolescent format) as mock protection from COVID-19, with the (re)posters speaking the same humorous voice for the sake of joint entertainment. The memes deploy, presumably, the very same CD image through the use of picture-editing software, as corroborated by the fact that the reflections on the silver surfaces are identical in all the memes and the rubber lines seem to have been drawn, except for the bearded man’s image. While in most cases, the meme authors will be the subjects themselves or their collaborators; in others, unknowing individuals’ images may be deployed, as is – presumably – the case with the elderly woman.

**Figure 9.**
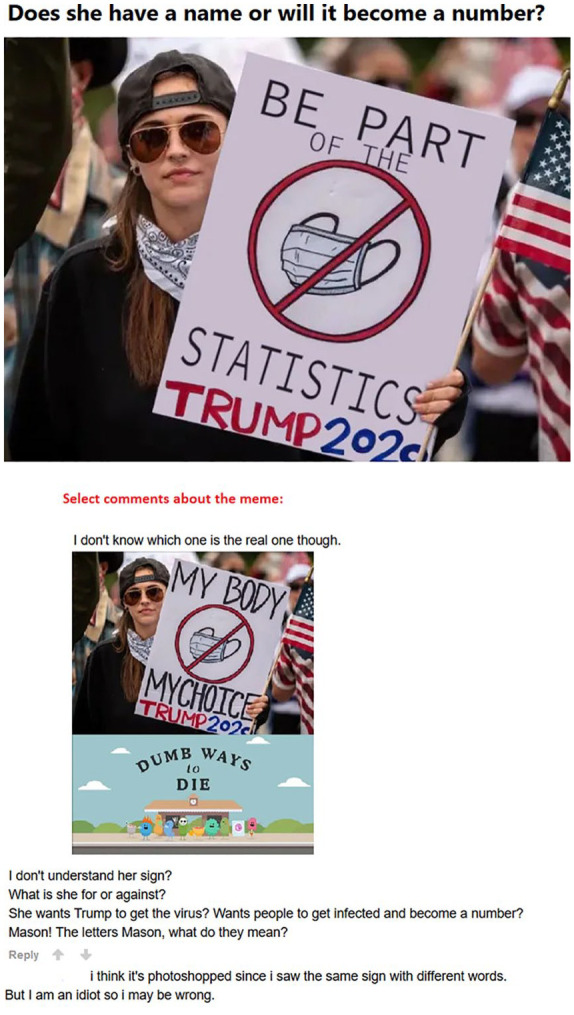
Multiplicity of voices in a confusing meme.

The multiplicity of nested voices and their epistemological ambiguity, also evidenced by metapragmatic comments from confused users, is most pronounced in a meme which does not subscribe to any of the main trends found in the corpus (see Figure 9).

The meme in Figure 9 is followed by users’ metapragmatic discussion, including a repost of a related meme. This first multimodal comment indicates the recognised voices involved in the construction of the meme at hand, which is indeed anchored in a manipulated version of the original viral photograph. This photograph shows a woman in Texas taking part in a protest rally against the state’s stay-at-home order.^12^ She is holding an intertextuality-based sign echoing the voice of those fighting for reproductive freedom, ‘My Body, My Choice’. While quoting this motto, the subject transposes a new meaning onto it by refusing to wear a face mask, as evidenced by not only the crossed-out mask drawing but also her bare face (with a bandana under her chin). As her sign also indicates, in doing so, she approvingly echoes President Trump’s public statement from 3rd April 2020 that masks should be worn on a ‘voluntary’ basis. The meme reposted in the comment combines this non-humorous photograph of the subject (multimodally reporting her voice) with a critical intertextual commentary. This involves the ‘Dumb Ways to Die’ poster, an Australian public service announcement campaign made by Metro Trains in Melbourne to promote railway safety, which went viral on social media in November 2012. Representing a critical voice similar to the meme in the comment, the main meme dissociatively echoes the voice of the subject, whose sign (cf. ‘Be part of the [no mask] statistics’) has been manipulated through picture-editing software. Coupled with the header, this meme voices a non-humorous opinion that not wearing a mask can turn the protesting subject into one of the numerous coronavirus victims. This meme, which draws on different voices and intertextuality, can be fully understood only thanks to a thorough investigation of the micro- and historical macro-context, the type of analysis to which many memes are not amenable.

## Discussion and final comments

The data systematically culled from four social media platforms present interesting empirical findings about COVID-19 mask memes, one topic of memes amid the 2020 pandemic. The analysis of early 2020 memes has yielded several salient memetic trends, most of which involve meme subjects. These trends are: (1) the dissociative echoing of multimodally quoted meme subjects (butts) sincerely wearing homemade masks, (2) the dissociative echoing of the collective voice of mask-wearers through parodic imitation (including meme series, such as photographs of subjects with saucepan lids or photoshopped anti-virus software CDs) and (3) (non)parodic offline/online pranks reported on social media.

All these trends are indicative of the multiplicity of multimodal voices (heard through not only verbal but also multimodal discourse that merges visual and verbal components) involved in meme creation and circulation. Hence, this investigation inspires new theoretical generalisations about the different participatory roles in meme production, as well as the polyvocality of memes that do not reuse previous meme templates or stock images applicable across various topics but are based on novel images of human subjects, the images that can go viral or become meme templates themselves. This is an important contribution to humour research on memes in general.

The voices of subjects, authors and (re)posters (the different participant roles) may be in tune or out of tune. The meme subject and author may be one person or collaborators (whether or not this is evident in the meme), thus speaking one voice. Additionally, users who post similar memes or repost the very same (viral) meme may join an asynchronous choir, tacitly echoing one another in an approving manner. Alternatively, voices may diverge. A meme subject’s voice may be multimodally echoed verbatim in a dissociative manner, whereby it is ridiculed. On the other hand, dissociative echoing can also be done through parodic imitation of an unidentified collective voice. Such parody (Dentith, 2000; Hutcheon, 1985; Rossen-Knill and Henry, 1997; Vásquez, 2016, 2019 and references therein) does not need to involve any serious criticism or meanness towards the parodied voice or principles (e.g. the need to wear masks), being a matter of *autotelic* humour (Dynel, 2017, 2018), that is humorous play for its own sake (Bateson, 1972 [1955]; see also Dynel and Poppi, 2019).

Moreover, when memetic modifications are posted or when the same meme is reposted, that is replicated verbatim across social media with no additions (often going viral, as is the case with a few instances presented in the course of this paper), the voice of the meme subject/author/previous poster may be re-purposed. This can also be thought of as a shift of *stance*, namely ‘the ways in which addressers [posters] position themselves in relation to the text, its linguistic codes, its addressees, and other potential speakers’, and ‘when re-creating a text, users can decide to imitate a certain position that they find appealing or use an utterly different discursive orientation’ (Shifman, 2013: 40). Furthermore, meme (re)posters may also unwittingly distort the voice and stance of the subject and/or the author or the previous poster, misinterpreting their original intent. Thus, for instance, a parodic act or a spoof on the subject’s part is taken as an act of sincere mask-wearing. However, this misinterpretation may actually be purposeful and entail playful or innocuous deception (Dynel, 2018). Such re-purposing, which cannot be easily distinguished from inadvertent distortion, is tantamount to trolling, understood in the traditional sense (Dynel, 2016b and references therein). Thus, for entertainment purposes, playfully trolling (re)posters may feign naïveté and take subjects’ images at face value (typically, as presenting sincere protective measures) only to circulate them in line with this purposeful (mis)interpretation (see Dynel, 2018 on covert pretending to misunderstand) in the hope that receivers will follow this naïve interpretation.

Overall, memes about COVID-19 masks leave some room for misunderstandings, or simply multiple interpretations, about the subject’s and author’s voices from both user and academic perspectives. As the analysis of a few items has borne out, what appears to be parodic voices or spoofs may be mistaken for, and (re)posted as, sincere voices (and, potentially, vice versa: sincere acts could be deemed parodies). This is something that can be interpreted in the light of Poe’s Law: ‘Without a winking smiley or other blatant display of humor, it is utterly impossible to parody a Creationist in such a way that *someone* won’t mistake for the genuine article’.^13^ While this original comment (posted on a forum about Christianity) concerned creationist views, it is relevant to any fundamentalist or extremist voices, in this case, manifest in creative but desperate protection from the coronavirus, which some memes dissociatively report. Poe’s law is also often associated with another (older) maxim present on many non-academic websites (including Wikipedia and KnowYourMeme) and in academic publications (Milner, 2016: 142, 143). In 2001, a Google-groups user transformed Arthur C. Clarke’s well-known maxim for science-fiction writers (‘Any sufficiently advanced technology is indistinguishable from magic’) into a ‘law’ for the Internet known as ‘Alan’s 2nd Law of Newsgroups’: ‘Any sufficiently advanced troll is indistinguishable from a genuine kook’.^14^ This, in turn, can be developed into ‘Any sufficiently advanced parody is indistinguishable from a genuine kook [their product]’ (another adage misattributed to the user by the name of Alan). These last two principles do apply to some of the data at hand, for it is impossible to conjecture, let alone unequivocally determine (even through a consistent online search), many a voice, which may be serious (indicative of a sincere act of mask-wearing) or purely humorous (e.g. parody done for fun). It may also involve some deception on the subject’s, author’s and/or poster’s side, so that receivers should believe that the representation is genuine, rather than being fabricated. Needless to say, these observations about the epistemological complexity and ambiguity of memes cannot be restricted to COVID-19 mask memes, which is what humour researchers are advised to bear in mind as they are studying their memetic data.

Just like the true nature and repercussions of COVID-19, the voices underlying memes are veiled in mystery. While humanity will, hopefully, fully understand and inactivate the virus, online users’ voices behind their humorous memetic produce can never be established beyond any doubt.

## Notes

1. For example https://thefunnybeaver.com/21-funny-corona-memes/
https://www.insider.com/coronavirus-memes-people-joking-about-covid-19-to-reduce-stress-2020-3

https://www.theatlantic.com/international/archive/2020/04/humor-laughter-coronavirus-covid19/609184/

https://www.deadlinedetroit.com/articles/25078/covid_jokes_aren_t_quarantined_‒_see_what_9_people_post_for_comic_relief

2. These examples, as well as those in the corpus, have been submitted by users with a view to being publicly available and can be accessed without signing in; therefore, selected manually and anonymised, such examples can be freely used for academic purposes in line with the standard ethical practice (Townsend and Wallace, 2016; cf. franzke et al., 2020).

3. In literary-theoretic terms, this notion also refers to how the voices of characters allow a novelist to portray ‘specific points of view on the world’ paving the way for ‘the orchestration of his [or her] themes and for the refracted (indirect) expression of his [or her] intentions’ (Bakhtin, 1981 [1975]: 292).

4.
https://www.nature.com/articles/d41586-020-02801-8

5. With the benefit of hindsight, it should be noted that memes collected in the second part of the year would – presumably – have shown the growth of different trends, such as memes chastising people who refuse to wear masks or who wear them inappropriately.

6. Formally, the photographer does not need to be the meme author (the person to publicise the picture online).

7. Between the 17th and 19th centuries, some plague doctors wore such masks for protection from putrid air, which was wrongly – according to the state-of-the-art knowledge we have – believed to be the cause of the illness.

8. For example https://www.reddit.com/r/photoshopbattles/comments/fm4fv5/psbattle_this_plague_doctor_buying_corona/

9. Another relevant case is a selection of memes based on copyrighted images taken from a project entitled *How To Survive A Deadly Global Virus* and created by Max Siedentopf, a designer based in London. This provocative project, allegedly serious and inspired by people’s homemade masks, features a series of photographs that present everyday items (e.g. a Nutella-jar mask, a cabbage leaf with holes for eyes, and a training shoe) being used as face masks. Meme posters have taken the pictures of some of Siedentopf’s models – whether or not aware of their provenance and the intertextuality – and reposted them as genuinely worn masks.

10.
https://www.tobaccocontrollaws.org/legislation/country/china/summary

11.
https://imgflip.com/memetemplate/220294056/Outbreak-smoke-brake

12.
https://rewire.news/article/2020/04/22/the-rights-desperate-attempts-to-hijack-my-body-my-choice/

13.
https://www.christianforums.com/threads/big-contradictions-in-the-evolution-theory.1962980/page-3#post-17605750

14.
https://groups.google.com/forum/?fromgroups=#!topic/talk.origins/r7ksugaCs8c%5B1-25%5D
